# Predictors of nursing leadership in Uganda: a cross-sectional study

**DOI:** 10.1093/heapol/czaa100

**Published:** 2020-11-09

**Authors:** Rose Clarke Nanyonga, Edna N Bosire, David J Heller, Elizabeth Bradley, Nancy R Reynolds

**Affiliations:** c1 Clarke International University, 3rd Floor, International Hospital Kampala Building, Kampala, Uganda; c2 South African Medical Research Council Developmental Pathways for Health Research Unit (DPHRU), School of Clinical Medicine, Faculty of Health Sciences, University of the Witwatersrand, Johannesburg, South Africa; c3 Icahn School of Medicine at Mount Sinai, Arnhold Institute for Global Health, New York, NY, USA; c4 Vassar College, Poughkeepsie, NY 12604, USA; c5 Johns Hopkins School of Nursing, 525 N. Wolfe Street, Baltimore, MD 21205, USA

**Keywords:** Nurses-in-leadership, leadership practices inventory, followers, organizational culture, Uganda

## Abstract

Evidence regarding the role of nurses-in-leadership and how to engage nurses in policy decisions is minimal in sub-Saharan Africa. The purpose of this study was: (1) to assess the leadership practices of nurses-in-leadership in Uganda (by self-report) and from the perspective of ‘followers’ (direct-report, peers, co-workers, other); and (2) to determine factors (positively) associated with leadership practices. We surveyed 480 nurses, 120 in leadership roles (*Response Rate* 57%) and 360 ‘followers’ (*Response Rate* 60%), who were recruited from five hospitals in Kampala, Uganda. We used the Leadership Practice Inventory (Self and Observer), a project-specific demographic questionnaire and Denison’s Organizational Culture Survey (DOCS). Sixty-three per cent of the respondents held a registered nursing certificate; 79% had received formal leadership training; 47% were based in private for-profit (PFP) hospitals, 28% in private not-for-profit (PNFP) and 25% in public hospitals. Among the five leadership practices, nurses-in-leadership used the practice of *Model the Way* (M *= *8.27, SD* = *1.30), C*hallenge the Process* (M *= *8.12, SD* = *1.30) and *Encourage the Heart* (M* = *8.04, SD* = *1.51) more frequently (on a 10-point Likert Scale). *Inspire a Shared Vision* (M *= *7.82, SD* = *1.57) and *Enable Others to Act* (M* = *7.62, SD* = *1.66) practices were used less frequently*.* The same rank order was true for leadership scores from the perception of followers. However, leadership scores by followers were significantly lower (*P *<* *0.01) than the nurse leader self-reported scores across all sub-scales. Leadership practice scores were higher in public than private hospitals (*P *<* *0.0001). Organizational culture (OC) was associated (*P *<* *0.001) with leadership practices. Although overall leadership practice scores were generally high, the less frequent use of *Inspire* and *Enable* practices suggests opportunities for targeted improvement. Moreover, differences between self-reported and leadership scores by followers suggest perception gaps between leaders and their followers. The positive relationship between public hospital settings and self-reported leadership practices among nurses-in-leadership suggests that important nursing leadership practices are possible even in a low-resource clinical setting.

**KEY MESSAGES**

- Nurses-in-leadership in low-income countries possess transformational leadership practices of highly successful leaders: model the way, inspire a shared vision, challenge the process, enable others to act and encourage the heart.
- Despite these skills, a significant number of nurses-in-leadership are often excluded from policy decisions, albeit in the absence of appropriate leadership appraisals.
- Leadership contexts that encourage personal growth, involvement in day-to-day decisions, a sense of direction and clear performance expectations enable nurse leaders to inspire others, lead change initiatives, foster collaboration, strengthen others and yield greater leadership outcomes.

## Introduction 

Health system failures in sub-Saharan Africa (SSA) are due in part to weak or absent leadership capacity (Mbacke, 2013; Omaswa and Crisp, 2014; Ezeh, 2015). Despite marked improvements, the region’s health systems continue to struggle to meet basic standards of care. Health system redesign and capacity-building are now key features of the agenda for health systems strengthening, and a critical component to combating human resources for health crises (HRHC) in low-to-middle-income countries (Chen *et al.*, 2004; Mbacke, 2013; WHO, 2016a). Recommended strategies prioritize leadership as a key strategy for effective service delivery and successful health system redesign (Chiarella, 2007; O'Neil, 2008; Daire *et al.*, 2014). A fundamental strategy is the inclusion of nurses, the most numerous of health care providers, in reform efforts. In light of the perceived weaknesses and looming crisis of nursing leadership (WHO, 2016b; Gwebu, 2017; Lamb *et al.*, 2020), international organizations including the Global Nursing Now Campaign (Crisp and Iro, 2018a; NNC, 2019), the Triple Impact Report (APPG, 2016), the International Council of Nursing (ICN, 2019) and the World Health Organization’s global strategy on Human Resources for Health: Workforce 2030 (WHO, 2016c) have prioritized strengthening nursing leadership and management capacity as a key strategy to counter the impact of HRHC on the health system and improve quality of life for patients globally. More recently, the WHO State of the World’s Nursing (SOWN) Report 2020 (WHO, 2020) has also called upon countries to prioritize investments in nursing leadership along with improved education and jobs.

Both the SOWN report and the ICN 2017–20 strategic priorities position nurses as an important group for policy-setting, decision-making and implementation of national and international policies (ICN, 2019; WHO, 2020). However, the critical shortage of other health care workers in SSA (Anyangwe and Mtonga, 2007; Scheffler *et al.*, 2009; Mullan *et al.*, 2011) creates a cascade of leadership needs, shapes the context in which leadership occurs and places greater demands on nurses-in-leadership (those in positions of leadership through assigned or emergent leadership roles) to motivate, encourage and challenge an otherwise overburdened, overstressed and unmotivated workforce (Anyangwe and Mtonga, 2007; Connell *et al.*, 2007). HRHC are most prevalent among nurses, and the leadership shortage is reported as more acute among the nursing workforce (Zuyderduin *et al.*, 2010; Zittel *et al.*, 2012). Nurses-in-leadership are particularly challenged by the rigour of leadership demands placed on them, as most health systems need leaders who are able to operate in contexts facing high disease burden and systemic challenges, compounded by inadequate investment in health systems (Zittel *et al.*, 2012; Premji and Hatfield, 2016; Gwebu, 2017). Considerable emphasis is placed on developing strategies for the efficient use of often a diminished pool of human and other resources by fostering change, innovation and resourcefulness and enlisting and mobilizing group (leaders and followers) actions towards a shared vision (Kirigia and Barry, 2008; Pillay, 2008). Nurses-in-leadership are also expected to demonstrate leadership skills suitable for attracting and retaining nurses, developing evidence-based programmes for equipping future leaders, while responding effectively to emerging health system challenges (Munjanja *et al.*, Dovlo, 2005; Premji and Hatfield, 2016; Crisp and Iro, 2018b; Rosa *et al.*, 2019).

Inclusion of nurses-in-leadership in policy discussions is paramount for prompting effective innovation in health care practice (ICN, 2019; WHO, 2020). The majority of nurses-in-leadership are well-placed in the organizational hierarchy for linking front-line staff to middle and senior management and leadership roles—by communicating, coordinating, championing and pioneering change (Banaszak-Holl *et al.*, 2011). Thus, nurses represent an untapped leadership resource in health systems seeking to develop stronger and more effective leaders. Advocates of inclusion of nurses-in-leadership in health system design argue for a paradigm shift—from functional doers, to proactive organizational leaders in health systems strengthening (Benton, 2012; Crisp and Iro, 2018b; WHO, 2020).

The effectiveness of nurses-in-leadership is dependent on essential transformational leadership practices (TLP) (Cowden *et al.*, 2011; Ross *et al.*, 2014). Transformational leadership is a style of leadership that inspires and enables followers to achieve extraordinary results, and helps leaders align the objectives and goals of the followers, the leader, groups and the organization to foster personal and organizational change (Ross *et al.*, 2014). Studies (Taurangaeu, 2004; Wong and Cummings, 2007; Porter-O’Grady, 2009; Clavelle *et al.*, 2012; Ross *et al.*, 2014) indicate that nurses-in-leadership who adopt the tenets of TLP, e.g. have the capacity to: (1) Lead by example (*Model*); (2) Inspire, innovate and communicate their vision (*Inspire*); (3) Strive for change and cultivate effective practice environments (*Challenge*); (4) Empower and move followers towards a common goal (*Enable*); and (5) Renew the spirit (*Encourage*); and hence (6) Have the potential to shape patient, staff and organizational outcomes.

As such, TLP are pivotal for the role of nurses-in-leadership in SSA to challenge the traditional ways of thinking, pioneer change, as well as establishing a practical evidence-based guide for the development of future leaders (Tourangeau *et al.*, 2010; Curtis *et al.*, 2011; Khoury *et al.*, 2011). However, few studies (Curry *et al.*, 2012; Posner, 2013, 2016) have evaluated the leadership practices of health care providers in SSA. Studies evaluating the leadership practices of nurses specifically have been limited to the USA and Canada (Tourangeau and McGilton, 2004; Porter-O’Grady, 2009; Clavelle *et al.*, 2012; Fardellone *et al.*, 2014; Ross *et al.*, 2014). The looming nursing leadership crisis in SSA (Gwebu, 2017) offers nurses-in-leadership a wide agenda for action, and underscores the need to understand the level of leadership preparedness as well as elements of what constitutes a leadership-enabling environment for nurses-in-leadership in SSA.

## Country profile: Uganda

Uganda is no exception to workforce challenges, and therefore offered an ideal case with which to examine the leadership practices of nurses-in-leadership. For example, nursing and midwifery practice in Uganda occurs in the context of increasing disease burden, including communicable diseases, non-communicable diseases (including mental health disorders), maternal and perinatal conditions, as well as neglected tropical diseases (WHO, 2018). In addition, declining human resources, poor working environments, a lack of leadership capacity and a general lack of adequate healthcare infrastructure remain a challenge (Barugahara *et al.*, 2008; Kyakuwa, 2011; Jaskiewicz *et al*, 2016; Madinah, 2016). Against this backdrop, the country has made substantial strides towards health systems rebuilding through a series of health sector reforms launched in 1994 (MoH, 2010). Key reform objectives that have had a direct impact on the re-organization of the nursing workforce include: creating positive practice environments as a retention strategy designed to address the loss of skilled professionals in developed countries; a renewed focus on strengthening the regulatory frameworks of professional councils; health system redesign with greater emphasis on decentralization and basic health care packages through strengthening primary health care; ensuring good governance and leadership; and improving access to and quality of services provided (Matsiko, 2010; MoH, 2010, 2016).

Uganda’s efforts towards health systems strengthening are laudable. However, although this study was conducted between 2013 and 2014, a critical gap still exists in the paucity of evidence to support the current calls for investments in nursing leadership capacity. An extensive literature search on studies examining leadership among nurses in Uganda yielded only two studies on leadership. Ziegler *et al.* (1997) assessed nurse leadership in community centres and documented challenges associated with nursing leadership roles, such as lack of formal job descriptions; an increase in responsibility without an increase in authority; and lack of leadership training, mentoring and support. Shariff (2014) examined the leadership attributes necessary for national nurse-leaders’ participation in health policy development in Uganda, Kenya and Tanzania. Results indicated that the essential leadership attributes for nurse-leaders’ participation in health policy included their ability to influence, communicate effectively, build relationships, feel empowered and demonstrate professional credibility. Yet, a significant number of nurses-in-leadership are still excluded from policy decisions affecting their practice or lack opportunities to participate meaningfully in health care decision-making processes and policy (Oulton, 2006; Chiarella, 2007; Shariff, 2014).

Current policy interventions as a result of the NNC-Uganda (NNC-Uganda, 2019) and the first Uganda National Nursing and Midwifery Policy (MoH, 2016), both of which prioritize the urgent need for effective nurses-in-leadership at clinical, organizational and national levels, do so in the absence of appropriate leadership assessment in context. Uganda’s NNC road map, in particular, focuses on three critical areas of transformation: Nursing Practice; nursing education and research; and nursing regulation. These are all underpinned by a need to foster nursing leadership capacity (NNC-Uganda, 2019). Hence, findings from this study remain relevant to inform this gap and support evidence-based action.

The nursing workforce accounts for 80% of the health workforce in Uganda, which includes, nursing, midwifery, medical doctors, allied health practitioners and traditional medical practitioners (MoH, 2019), thus their role and the scope of their practice continue to be of paramount importance to the success of health care reforms. However, the density ratio of nurse/midwife to patients has remained at 0.63 (2015) per 1000 people (World Bank, 2019), indicating minimal growth in numbers despite increasing demands on the nursing workforce. Furthermore, 70% of the nursing workforce are certificate holders with limited capacity to provide evidence-based practice or participate meaningfully in health care reforms. The majority (2%) of the highly educated nurses work outside of the hospital setting, which may limit their involvement in the hospital policy agenda and their ability to foster change (NNC, 2019). This gap is one of the primary focuses of the NNC-Uganda's Florence Nightingale Challenge (NNC, 2019) which aims to train >200 nursing leaders in Uganda. It highlights the need to increase the number of available nurses-in-leadership, and evaluates the ability, quality and effectiveness of the current and future nursing leaders in the country.

Accordingly, this study’s aims were:

1. To describe the current level of leadership practices [Model the way (Model), Inspire a shared vision (Inspire), Challenge the process (Challenge), Enable others to act (Enable) and Encourage the heart (Encourage)] of nurses-in-leadership in hospitals in Uganda from two perspectives: 1a, nurses-in-leadership self-reported leadership practices Leadership Practice Inventory (LPI-Self); and 1b, leadership practices of nurses-in-leadership from the perspective of their followers (those who routinely interact with and observe them, including direct reports, peers and others) (LPI-Observer).
2. To identify correlates of self-reported leadership practices, particularly with factors related to demographics (Age, Gender, Level of Education, Leadership Training, Tenure, Rank and Hospital Type) and OC (Involvement, Consistency, Adaptability and Mission) to identify tools to bolster nursing leadership in Uganda and other low-resource settings.

**Figure 1:**
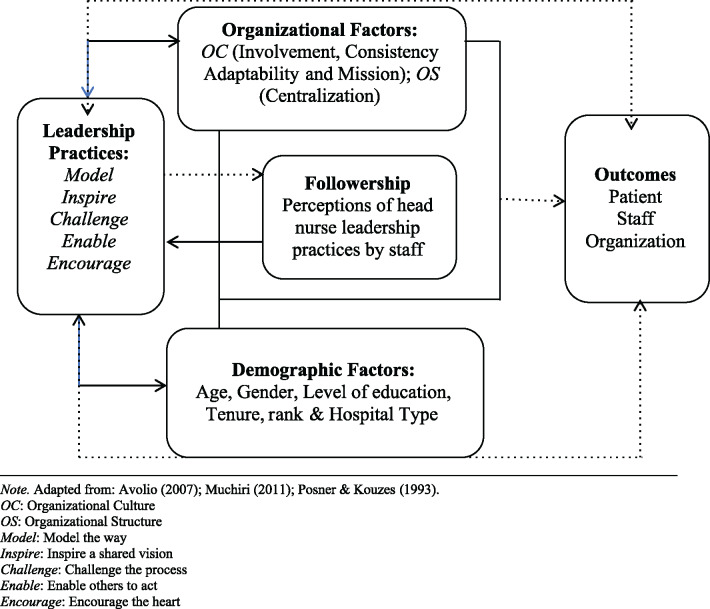
Integrated model of [nursing] leadership. Adapted from: Avolio (2007), Muchiri (2011) and Posner and Kouzes (1993). *OC*, organizational culture; *OS*, organizational structure; *Model*, model the way; *Inspire*, inspire a shared vision; *Challenge*, challenge the process; *Enable*, enable others to act; *Encourage*, encourage the heart

The Integrated Model of [Nursing] Leadership guided this study and reflects a conceptualization of leadership inclusive of leaders, followers and the context (Avolio, 2007; Avolio *et al.*, 2009; Muchiri, 2011) (see Figure 1). Leadership, as proposed by House *et al.* (2004), is the ability of an individual to influence, motivate and enable others to contribute towards the effectiveness and success of the organization of which they are a part. The model represents a synthesis of leadership practices, leader-follower interactions (perceptions), as well as contextual boundaries of leadership. Integrative perspectives view leadership as a group quality, i.e. as a set of functions that must be carried out by the group (leaders and followers) within a specific context (Gronn, 2000). This approach calls for a recognition of the emergent nature of leadership processes and the contextual environment within which these processes occur (Avolio, 2007). Contexts can be thought of as a platform that facilitates the shaping and frequency of leadership practice and allows for a dynamic relationship to exist between organizational context, leaders and followers (Hargrove and Owens, 2002; Volckmann, 2005; Laschinger *et al.*, 2009). Evidence also suggests that demographic variables (such as age, gender, level of education, tenure and rank) play an important role in the quality of leader–follower exchanges (Lau *et al.*, 2008). Thus, identifying the factors associated with leadership practices provides an understanding of how demographics and contextual variables function as potential parameters of leadership processes and practice.

## Methods

### Study design

We conducted this descriptive, cross-sectional study between August 2013 and February 2014. Participant recruitment was based on accessible populations of nurses-in-leadership and staff from hospitals in Kampala who consented to participate in the study.

### Context

This study was conducted in hospitals within Kampala, where at least 58.4% of nurse and midwifery professionals, 40.6% of nurse and midwifery associated professionals and 30.2% of nurse assistants/aids are in urban centres (MoH, 2011). The target population for this study was the nursing workforce in Uganda. Study locations included publicly available listings of private for-profit (PFP), private not-for-profit (PNFP) and public hospital types. The public hospital, Mulago, is a national referral hospital, one of two in Uganda and the largest facility in the country. National referral hospitals provide comprehensive advanced tertiary care and specialist services, and are involved in teaching and research. The other study centres in Kampala were listed as general PFP (three hospitals) and PNFP (one missionary hospital) offering services at the district level. The estimated population served by general hospitals is 500 000. At least 61.5% of the nursing workforce is reported as working in the private sector.

### Study population and sampling procedure

**Figure 2:**
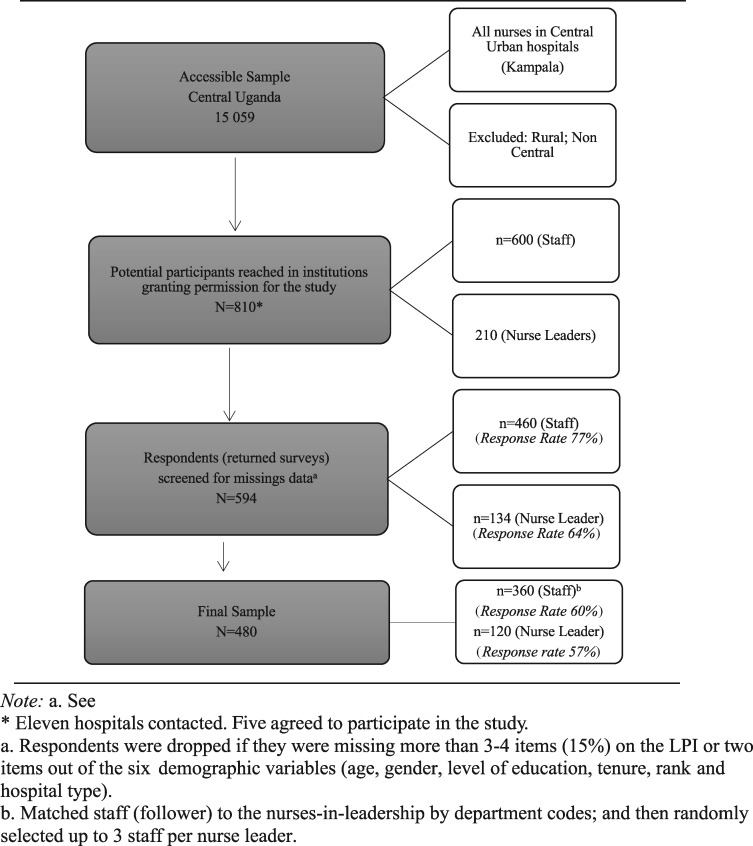
Sampling frame. *Eleven hospitals contacted. Five agreed to participate in the study. ^a^Respondents were dropped if they were missing more than three and four items (15%) on the LPI or two items out of the six the demographic variables (age, gender, level of education, tenure, rank and hospital type). ^b^Matched staff (follower) to the nurses-in-leadership by department codes; and then randomly selected up to three staff per nurse leader

We used a non-probability convenience sampling procedure to recruit study participants, due to the lack of a database to systematically select them. The sample was composed of the following: For research aim 1 and 2, all accessible nurses-in-leadership working in PFP, PNFP and public hospital types. For research aim 1b (assessing followers’ perceptions), all accessible staff-nurses working in the same unit as the nurse-in-leadership were eligible. Three to five staff-nurses corresponding to each nurse-in-leadership were chosen based on an average of 3–5 followers, which is recommended by the developers of the LPI-Self/-Observer (Posner and Kouzes, 1993) (see Figure 2). Six institutions (two public, two PNFP, two PFP) that did not respond to the investigators' request to access their facility for research purposes were considered ‘inaccessible’ and were excluded from the study.

The inclusion criteria were all available staff nurses (followers) and nurses-in-leadership who: worked full time in one of the three hospital types; were on duty at the time of data collection which was during the day shift (7 am–6 pm); and met the criteria of professional nurse (registered nurse/midwife/comprehensive [RN/RM/RCN], Enrolled nurse/midwife [EN/M]). Nurses-in-leadership included nurses with a formal title of: Senior Nursing Officers and their assistants, Area Managers, Heads of Department and assistants, Principal Nursing Officers and assistants, Charge Nurses, Chief Nursing Officers and assistants and Team Leaders or Nurse Educators.

Staff nurses (followers or observers) had to work on the same unit as the nurse leader, be on duty at the time of data collection and meet the following description: direct-report, peer, co-worker or other (observer).

### Data collection procedures and instruments

A master list of departments in participating facilities was generated prior to the distribution of survey packages, and was coded by department, participant and hospital type. Questionnaires were distributed to all available nurses willing to participate in the study at the beginning of a work shift (7 am) and collected before the end of the shift (6 pm).

Self-reported data were collected via three survey instruments: (1) Demographic questionnaire, (2) the LPI-Self and -Observer and (3) DOCS. Questionnaires were administered in hard copy in lieu of anticipated limited access to the electronic version of the measures.

#### Measures

Demographic data were collected using a project-specific demographic questionnaire. Data included: age, gender, level of education, leadership training, tenure (job, leadership, and organizational tenure), rank (leaders only) and hospital type (PFP, PNFP and public). Additional demographic data included formal position, and a classification of the staff nurses’ relationship to the nurse-in-leadership (whether the staff member directly reported to them, was their peer or was another type of observer).

Leadership practices were measured using the LPI-Self and -Observer, which measure five key practices of exemplary leadership—*Model*, *Inspire*, *Challenge*, *Encourage* and *Enable—*included in Kouzes and Posner’s Five TLP Framework (2003). According to Kouzes (2011), leadership practices reflect what individuals do repeatedly to develop as effective leaders. Each leadership practice constitutes a subscale with six behavioural statements (for a total of 30 items). Participants indicate the frequency to which they engage in the leadership practices on a 10-point Likert scale, with 1 indicating low frequency (almost never) and 10 indicating high (almost always) (see Table 1 for behavioural qualities that comprise leadership practices). The tool measures the extent to which a leader practises these behaviours.

**Table 1:** The leadership practices inventory: item description by sub-scale

Subscale	Item description
Modelling the way (model)	LPI1: Sets a personal example of what she/he expects of others LPI6: Spends time and energy making certain that the people she/he works with adhere to the principles and standards we have agreed on LPI11: Follows through on promises and commitments she/he makes LPI16: Asks for feedback on how his/her actions affect other people’s performance LPI21: Builds consensus around a common set of values for running our organization LPI26: Is clear about his/her philosophy of leadership
Inspire a shared vision	LPI2: Talks about future trends that will influence how our work gets done LPI7: Describes a compelling image of what our future could be like LPI12: Appeals to others to share an exciting dream of the future LPI17: Shows others how their long-term interests can be realized by enlisting in a common vision LPI22: Paints the ‘big picture’ of what we aspire to accomplish LPI27: Speaks with genuine conviction about the higher meaning and purpose of our work
Challenge the process	LPI3: Seeks out challenging opportunities that test own skills and abilities LPI8: Challenges people to try out new and innovative ways to do their work LPI13: I search outside the formal boundaries of my organization for innovative ways to improve what we do LPI18: Asks ‘What can we learn?’ when things don’t go as expected LPI23: Makes certain that we set achievable goals, make concrete plans and establish measurable milestones for the projects and programmes we work on LPI28: Experiments and takes risks, even when there is a chance of failure
Enable others to act	LPI4: Develops cooperative relationships between the people she/he works with LPI9: Actively listens to diverse points of view LPI14: Treats others with dignity and respect LPI19: Supports decisions that people make on their own LPI24: Gives people a great deal of freedom and choice in deciding how to do their work LPI29: Ensures that people grow in their jobs by learning new skills and developing themselves
Encouraging the heart	LPI5: Praises people for a job well done LPI10: Makes it a point to let people know about his/her confidence in their abilities LPI15: Treats others with dignity and respect LPI20: Publicly recognizes people who exemplify commitment to shared values LPI25: Finds ways to celebrate accomplishments LPI30: Gives members of the team lots of appreciation and support for their contributions

Copyright © 2013 James M. Kouzes and Barry Z. Posner. Published by John Wiley & Sons, Inc. All rights reserved.

Similarly, the LPI-Observer uses the same behavioural statements to assess the followers’ perception of the extent to which the leaders engage in practising these behaviours (Kouzes and Posner, 2003). Psychometric evaluation of the LPI was first conducted across a variety of public and private sector organizations using 2876 managers and their direct subordinates. Internal consistency reliability of the five scales ranged from *α*  =  0.70 to *α*  =  0.84 for the LPI-Self and *α*  =  0.79 to *α*  =  0.91 for the LPI-Observer. The average test–retest reliability from the original study was reported at *α*  = 0.94. Nursing leadership meta-reviews that have been conducted thus far rate the LPI among the most widely used measurement tools of leadership practices (Cummings *et al.*, 2008). Posner (2013) also used the tool to evaluate and compare the leadership practices of health care providers in India, Pakistan, Ethiopia and the Philippines.

OC was measured by DOCS (Denison *et al.*, 2006). The tool is a reliable and valid instrument that has been used in similar settings as with the LPI (Cummings *et al.*, 2008; Jung *et al.*, 2009). The assessment measures to what extent an organization is perceived to display the four dimensions of characteristics that influence leadership and organizational performance: (1) *Involvement*—by staff and leaders in the day-to-day tasks and environment where employee involvement in decision-making and a sense of ownership are highly encouraged; (2) *Consistency*—of organizational procedures within an organization that creates internal systems of governance based on consensual support; (3) *Adaptability*—the organization’s ability to translate the demands of the customer, practice environment and external markets into action; and (4) *Mission*—the organization’s ability to define a meaningful long-term direction and share a common vision for the future (see House *et al.*, 2004; Zagorsek *et al.*, 2004; Denison *et al.*, 2006; Casida and Pinto-Zipp, 2008). Respondents describe their OC using a five-point Likert scale (1 = strongly disagree, 2 = disagree, 3 = neutral, 4 = somewhat agree and 5 = strongly agree). Consistency reliability tests were performed for both tools for the current study. The total LPI-Self had high internal consistency reliability (*α*  =  0.95), as did the total LPI-Observer (*α*  = 0.97). In addition, the total reliability scale score for the DOCS was 0.92 (see Table 2).

**Table 2:** Internal consistency reliability of the LPI, DOCS

Measurement scale	No. of items	Sample (*N*)	*α* Coefficients Current study	*α* Coefficients Kouzes and Posner	
Total LPI (nurse-leaders) *Subscales*	30	120	0.95		
				Study^a^	Study^b^
Model the way	6		0.74	0.77	0.71
Inspire a shared vision	6		0.83	0.87	0.80
Challenge the process	6		0.73	0.80	0.70
Enable others to act	6		0.82	0.77	0.75
Encourage the heart	6		0.82	0.87	0.85
Total LPI (observers) *Subscales*	30	360	0.97		
Model the way	6		0.86	0.86	0.82
Inspire a shared vision	6		0.88	0.92	0.88
Challenge the process	6		0.85	0.89	0.81
Enable others to act	6		0.84	0.88	0.86
Encourage the heart	6		0.88	0.92	0.92
Total DOCS (nurseleaders) *Subscales*	60	120	0.92		
Involvement	15		0.77		
Consistency	15		0.81		
Adaptability	15		0.76		
Mission	15		0.77		

Comparison scores are from the following studies:

a Posner and Kouzes (1993): Psychometric properties of the LPI-updated.

b The IPL—theory and evidence behind the five practices of exemplary leaders (Kouzes and Posner, 2002).

### Ethical considerations

This study was approved by the Yale Human Subjects Committee. Institutional approval to carry out data collection and have access to the health facility and employees was obtained from the hospital administrator or equivalent authority at participating facilities. Participants received an introductory cover letter explaining the purpose of the study, and return of the questionnaires reflected informed consent. Participants’ names were anonymized for privacy.

### Data analysis

Data were analysed using the Statistical Package for the Social Sciences (SPSS) IBM version 22. Descriptive statistics were used to describe the demographic characteristics of the respondents (see Table 1). Internal consistency reliability for measures was assessed according to threshold values outlined by George and Mallery (2010): *α*  >  0.9 = excellent reliability, *α* 0.8 < *α*  <  0.9 = good reliability, *α* 0.7 < *α* <  0.8 = acceptable reliability, *α*  >  0.6 < *α* <  0.7 = questionable reliability, *α* 0.5 < *α* <  0.6 = poor reliability and *α* ≤ 0.5 = unacceptable reliability. Inter-total item correlations for the LPI were assessed as acceptable using Pallant and Manuals’ (2010) threshold of 0.30 or greater. Descriptive statistics were used to describe the self-reported leadership practices among nurses-in-leadership. Analysis of means and standard deviations was carried out. Leadership practice scores were calculated by averaging items that correspond with each subscale on the LPI. *Model* was calculated by averaging items 1, 6, 11, 16, 21 and 26. *Inspire* was calculated by averaging items 2, 7, 12, 17, 22 and 27. *Challenge* was calculated by averaging items 3, 8, 13, 18, 23 and 28. *Enable* was calculated by averaging items 4, 9, 14, 19, 24 and 29. And *Encourage* was calculated by averaging items 5, 10, 15, 20, 25 and 30. Mean scores ranged from 1 to 10, where higher scores indicate frequently used or observed leadership practice, and 1 indicates least frequently used or observed leadership practice. Spearman’s Rank Order correlation analysis was carried out to assess the relationship between nurse leaders self-reported scores and leadership scores of the nurse leader by followers. Cohen’s standard (Cohen, 2013) was used to evaluate the correlation coefficient to determine the strength of the relationship, where coefficients between 0.10 and 0.29 represent a small association, coefficients between 0.30 and 0.49 represent a medium association and coefficients >0 0.50 represent a large association. An additional analysis for inter-rater reliability (the degree to which different observers give consistent estimates of perceived or observed leadership practices) (Polit and Beck, 2008) was also calculated using a mixed effect model to generate the intra-class correlation coefficient (ICCs). Paired sample t-tests were carried out to assess differences in scores. Bivariate and multivariate analysis was carried out to assess factors associated with self-reported leadership practices. The dependent variables in the regression analysis included the five subscales of the LPI (*Model*, *Inspire*, *Challenge*, *Enable* and *Encourage*). The independent variables included the subscales of OC (*Involvement*, *Consistency*, *Adaptability* and *Mission*). The only control variable in the analysis was hospital type, which was the only demographic variable that was significantly associated with self-reported leadership practices from prior analysis.

## Results

### Demographic factors

Of the returned questionnaires, 120 nurses-in-leadership (Response Rate 57%) and 360 followers (Response Rate 60%) completed the survey, reflecting an average of three respondents for each nurse-leader. Of these, 87 (73%) and 274 (76%), respectively, were between the ages of 25 and 45 years. Participants were primarily female (99 [83%] nurses-in-leadership and 94 [82%] followers). Across the sample, 76 (63%) of nurses-in-leadership and 183 (51%) followers held an RN/RM/RCN certificate as their highest level of education, while <9% held a bachelor’s degree in nursing (BSN) or higher degrees for both groups. More than half (59%) of nurses-in-leadership were designated as ‘Head Nurse’, while 65% of followers identified as ‘General Staff Nurse’. The relationship to the nurse-in-leadership was classified into three categories: followers who had a directly reporting relationship to the leader (*n* = 252, 70%), followers who were peers (P) with the leader (*n* = 89, 25%) and other observers (who were supervisors or bosses) (B) of the leader they were rating (*n *= 19, 5%) (see Table 3).

**Table 3:** Demographic characteristics of participants

Demographics	Nurse leaders *N *=* *120		Staff *N *=* *360	
Age (years)				
<25 years	6	5%	60	17%
25–35 years	55	46%	198	55%
36–45 years	32	27%	76	21%
46+ years	27	22%	26	8%
Gender				
Female	99	83%	294	82%
Male	21^a^	18%	66^a^	18%
Level of education				
Enrolled: nurse/midwife/comprehensive nurse	12	10%	101	28%
Registered: nurse/midwife/comprehensive nurse	76	63%	183	51%
Double registered (nurse and midwife)	21	18%	46	13%
BSN or higher	11	9%	30	9%
Other (clinical officers, theatre attendants, patient care coordinators)	6	2%		
Tenure				
Job tenure: years of clinical experience				
<5 years	35	29%	201	55%
6–10 years	35	29%	100	28%
11–15 years	25	21%	36	10%
16–20 years	5	4%	5	1%
21+ years	20	16%	18	5%
Leadership tenure: years of leadership experience				
<5 years	61	51%	89	25%
6–10 years	36	30%	48	13%
11–15 years	10	8%	10	3%
16–20 years	3	3%	2	1%
21+ years	10	9%	4	1%
Organizational tenure: years in the same hospital				
<5 years	62	52%	260	72%
6–10 years	25	21%	68	19%
11–15 years	17	14%	22	6%
16–20 years	7	6%	4	1%
21+ years	9	7%	6	2%
Rank (level of management)				
Lower-level management	45	38%		
Middle-level management	46	38%		
Senior management	29	24%		
Hospital type				
PFP	56	47%	168	47%
PNFP	34	28%	102	28%
Public (government)	30	25%	90	25%
Formal leadership training				
Yes	95	79%		
No	25	21%		
Formal position				
Chief/principal nursing officer (includes assistants)	11	9%	5	1%
Head of department	16	13%	10	3%
Senior nursing officer (includes assistants)	12	10%	24	7%
Unit manager/charge nurse or team leader	22	18%	77	21%
Staff nurse	59	49%	234	65%
Relationship to the nurse-in-leadership				
Direct report			252	70%
Peer			89	25%
Other			19	5%

Percentages may not total 100 due to rounding error.

### Leadership practices

Nurse-in-leadership self-reported leadership practice mean (SD) scores ranged from highest 8.27 (SD* = *1.30) to lowest 7.6 (SD* *=* *1.66). The most frequently used leadership practice was *Model*, 8.27 (SD* *=* *1.30) followed by *Challenge*, 8.12 (SD* *=* *1.30), *Encourage*, 8.04 (SD* *=* *1.51), *Inspire*, 7.8 (SD* = *1.57) and *Enable*, 7.6 (SD* *=* *1.66). Nurse-leader scores by followers adhered to the same rank order, with nurses-in-leadership receiving the highest mean scores for *Model*, 7.48 (SD* = *1.69) and *Challenge*, 7.4 (SD* = *1.66), but the lowest scores on *Inspire*, 7.3 (SD* = *1.81) and Enable, 7.15 (SD* = *1.70) respectively; however, in each category, the follower report is lower than the nurse-in-leadership reported score (see Table 4).

**Table 4:** Descriptive statistics of leadership practices, OC

Variable	*N*	*M*	SD	Rank
Self-reported leadership practices^a^	120			
Model the way		8.27	1.30	1
Inspire a shared vision		7.82	1.57	4
Challenge the process		8.12	1.30	2
Enable others to act		7.62	1.66	5
Encourage the heart		8.04	1.51	3
Observed leadership practices	360			
Model the way		7.48	1.69	1
Inspire a shared vision		7.31	1.81	4
Challenge the process		7.44	1.66	2
Enable others to act		7.15	1.70	5
Encourage the heart		7.41	1.74	3
OC^b^	120			N/A
Involvement		3.80	0.54	
Consistency		3.66	0.59	
Adaptability		3.65	0.57	
Mission		3.57	0.55	

Same rank order between nurses-in-leadership and followers. Top scores for *Model the way*, while lowest ranking on *Enable others to act*.

a Leadership practices inventory is a 10-point Likert scale.

b Organizational culture measure (OC) is a 5-Point Likert scale.

### Followers' perceptions

The results of the Pearson correlation indicated that there was a significant positive association between leaders' and followers’ scores (*r*s = >.37–<.45, *P *<* *0.01) (see Table 5). A follow-up analysis was carried out using paired t-tests to assess difference in scores. Follower scores were significantly less (*P *<* *0.01) than nurse-in-leadership scores for all subscales (see Table 6). Given that this study is the first to use the LPI-Observer for nursing groups in Uganda, an additional ICC was calculated using a mixed effect model to assess consensus (as a measure of inter-observer) reliability for the LPI-Observer. The results for *Model* (0.27), *Inspire* (0.24), *Challenge* (0.21), *Enable* (0.17) and *Encourage* (0.26) indicated that inter-rater reliability was fairly low.

**Table 5:** Spearman correlations between self-reported leadership and followers' perceptions of nurse-in-leadership LPs

Variables	Model (Staff)	Inspire (Staff)	Challenge (Staff)	Enable (Staff)	Encourage (Staff)
Model	**0.45** **	0.43**	0.44**	0.32**	0.45**
Inspire	0.44**	**0.46** **	0.44**	0.42**	0.47**
Challenge	0.45**	0.38**	**0.39** **	0.37**	0.40**
Enable	0.45**	0.46**	0.44**	**0.43** **	0.46**
Encourage	0.38**	0.34**	0.35**	0.27**	**0.37** **

*N* = 120 (nurses-in-leadership); *N* = 360 (staff).

Variables: Model, model the way; Inspire, inspire a shared vision; Challenge, challenge the process; Enable, enable others to act; Encourage, encourage the heart.

** *P* < 0.01.

**Table 6:** Paired sample t-test statistics for leadership practices by self- and staff-reported

Variable	Self-reported		Staff-reported		*P*
	*M*	SD	*M*	SD	
Model the way	8.27	1.30	7.48	1.21	0.000**
Inspire a shared vision	7.82	1.57	7.31	1.27	0.000**
Challenge the process	8.12	1.30	7.44	1.14	0.000**
Enable others to act	7.62	1.66	7.15	1.14	0.002**
Encourage the heart	8.04	1.51	7.41	1.24	0.000**

*N = *120 (nurses-in-leadership); *N = *360 (Staff).

** *P *<* *0.01.

### Demographics and organizational factors

To assess the association between demographic variables (*Age*, *Gender*, *Level of Education*, *Leadership Training*, *Tenure*, *Rank* and *Hospital Type*) and leadership practices (*Model*, *Inspire*, *Challenge*, *Enable* and *Encourage*), a one-way MANOVA was carried out*.* Only hospital type was significantly associated with self-reported leadership practices, suggesting that at least 23% of the variance (*η*^2^ = 0.233, *P *<* *0.01) in leadership scores was accounted for by the type of hospital in which nurses-in-leadership worked (see Table 7).

**Table 7:** Results of MANOVA for self-reported leadership practices by demographic variables

Dependent variable	Independent variable	*F*	Partial *η*^2^	Sig
Model Inspire Challenge Enable Encourage	Age	1.25	0.053	0.24
	Gender	0.87^a^	0.037	0.50
	Level of education tenure	0.96	0.041	0.50
	Job tenure	0.81	0.035	0.70
	Leadership tenure	0.92	0.039	0.57
	Organizational tenure	1.05	0.045	0.40
	Rank	1.31^a^	0.055	0.23
	Hospital type	6.93^b^	0.233^b^	0.000**
	Leadership training	0.45	0.019	0.81
	Leader to staff ratio	0.94	0.040	0.53

Dependent variables: Model, model the way; Inspire, inspire a shared vision; Challenge, challenge the process; Enable, enable others to act; Encourage, encourage the heart.

a Exact statistic.

b Pillai’s trace reported.

** *P* < 0.01. *N* = 120.

A *post hoc* analysis of the variances in scores by hospital type indicated that leadership scores for PNFP hospital types were significantly (*P *<* *0.001) lower than scores of nurses-in-leadership in both PFP hospital types and public hospital types for all subscales respectively (*P*s < 0.001) (see Table 8).

**Table 8:** *Post hoc* ANOVA for self-rated leadership practices by hospital type (PFP vs PNFP vs public)

	Hospital type			
	PFP	PNFP	Public	
Dependent variable	*M*	*M*	*M*	*P*
Model	8.47	7.15	9.18	0.000***
Inspire	7.68	6.89	9.13	0.000***
Challenge	8.13	7.22	9.14	0.000***
Enable	7.45	6.64	9.06	0.000***
Encourage	8.10	6.92	9.21	0.000***

*N *=* *120*.* Dependent variables: Model, model the way; Inspire, inspire a shared vision; Challenge, challenge the process; Enable, enable others to act; Encourage, encourage the heart.

*** *P *<* *0.001.

PFP, private for-profit; PNFP, private not-for-profit.

To assess factors associated with leadership practices, multiple linear regression analyses were carried out. The hospital type remained significant for all leadership practices (*P* = < 0.001). Nurses-in-leadership working in PFP hospital types tended to use *Model* less frequently than nurses-in-leadership in public hospital types (*B* = −0.96, *P* = < 0.001); and those working in the PNFP hospital type tended to engage in *Model* less frequently than nurses-in-leadership in both PFP and public hospital types (*B* = −2.12, *P =* < 0.001). This was also true for the subscales *Inspire*, *Challenge*, *Enable* and *Encourage* (see Table 9).

**Table 9:** Multiple linear regression with OC on self-reported leadership practices independent variables: OC

Independent variables	Model		Inspire		Challenge		Enable		Encourage	
	SE	*β*	SE	*β*	SE	*β*	SE	*β*	SE	*β*
PFP^a^	0.32	−0.37**	0.41	−0.66**	0.32	−0.55**	0.41	−0.66**	0.37	−0.53**
PNFP^a^	0.27	−0.74**	0.35	−0.73**	0.27	−0.73**	0.35	−0.73**	0.32	−0.75**
Involvement	0.25	0.19	0.32	0.15	0.24	0.29**	0.32	0.15	0.28	0.23^*^
Consistency	0.26	0.03	0.34	0.10	0.26	−0.03	0.34	0.10	0.30	−0.02
Adaptability	0.30	−0.22	0.38	−0.20	0.30	−0.17	0.38	−0.20	0.34	−0.09
Mission	0.28	0.20	0.36	0.29**	0.28	0.27^*^	0.36	0.29**	0.32	0.25^*^
*R* ^2^	0.40		0.39		0.41		0.39		0.40	
*F*	10.81**		10.30**		10.94**		10.30**		10.65**	

Overall model: *F*(7, 112).

^a^ Control variables: hospital type (private for-profit; private not-for-profit).

* *P *<* *0.05.

** *P *<* *0.001.

In addition, OC was a significant predictor of the frequency of use of leadership practices for all subscales: *Model* (*R*^2^ = 0.40, *P *<* *0.001); *Inspire* (*R*^2^ = 0.39, *P *<* *0.001); *Challenge* (*R*^2^ = 0.41, *P *<* *0.001); *Enable* (*R*^2^ = 0.39, *P *<* *0.001); and *Encourage* (*R*^2^ = 0.40, *P *<* *0.001). This suggests that at least 39–40% of the variance in leadership scores was attributable to the OC of the hospitals. Specifically, *mission subscale* scores were statistically significant and positively associated with *Inspire* (*B *=* *0.91, *P *=* *0.009) and *Enable* (*B *=* *0.88, *P *=* *0.016), indicating that improvements in *mission* culture scores corresponded with an increase in frequency of use of *Inspire* and *Enable* leadership practices in all hospital types. In addition, *Mission scores* (*B *=* *0.64, *P *=* *0.023) and *Involvement scores* (*B *=* *0.71, *P *=* *0.004) were statistically significant and positively associated with *Challenge*, indicating that improvements in *mission* and *involvement* cultures correlated with frequent use of *Challenge* leadership practices. Similarly, *Involvement* (*B *=* *0.64, *P *=* *0.027) and *Mission* (*B *=* *0.68, *P *=* *0.037) were also significantly positively associated with *Encourage*, suggesting that increases in *involvement* and *mission* culture scores corresponded with an increase in frequency of use of this leadership practice.

## Discussion

Results of this study indicate that the nurses-in-leadership in Uganda perceived themselves as engaging in leadership behaviours that were consistent with the TLP that nurse leaders in other countries use to achieve extraordinary results: *Model the way*, *Inspire a shared vision*, *Challenge the process*, *Enable others to act* and *Encourage the heart*. The top three leadership practices reported in this study were *Model the way*, *Challenge the process* and *Encourage the heart*. *Model the way* relates to the leader’s ability to implement methods for leadership through role modelling, creating an environment for mentoring and fostering consensus around shared values (Clavelle *et al.*, 2012). *Challenge the process* leadership practices shifts the leaders’ orientation from the cultivation of self-insight and setting examples to making appropriate considerations for the participative engagement of others in challenging the process (Kouzes, 2011). And *Encourage the heart* leadership practices include the leaders’ ability to foster a culture of recognition, inspire confidence and celebrate team and individual accomplishments (Ross *et al.*, 2014). In the context of threats that substantially impact leadership in SSA, enabling the leadership talent of nurses-in-leadership who can model, inspire and challenge and who are confident in their ability to pioneer change initiatives may contribute favourably towards the broader goals of leadership capacity-building and health systems strengthening. All three of the top leadership practices are a key finding for organizations severely impacted by HRHC resulting in a disheartened, overburdened and overstressed workforce (Anyangwe and Mtonga, 2007; Connell *et al.*, 2007).

Our findings are consistent with other studies that have examined the TLP of nurses-in-leadership outside SSA, but with some noticeable differences. For example, Chief Nursing Officers in Magnet Hospitals in the USA reported, in rank order, *Enable*, *Model* and *Challenge* as the top three practices (Clavelle *et al.*, 2012). Porter-O’Grady’s (2009) study comparing Chief Nurse Executive TLP in Magnet and non-Magnet Hospitals identified *Enable*, *Model* and *Encourage* as the top three practices. Meanwhile, Ross *et al.* (2014) reported the top three TLP of nursing leaders in Professional Nursing Associations as *Enable, Encourage* and *Model* (see comparison in Table 10). Nurses-in-leadership in this study matched the three studies in ranking on *Model*, two studies on *Encourage* and one study on *Challenge* as top TLP, but with slightly lower scores across all subscales. The comparison with nurses-in-leadership in Magnet Organizations is particularly encouraging. Magnet Hospitals are classified by the American Nurses Credentialing Center (ANCC) as centres of excellence that embody Forces of Magnetism (attributes that exemplify a professional environment guided by visionary nursing leadership, and excellence in nursing practice) (ANCC, 2020).

**Table 10:** Comparison of leadership practices with previous nursing leadership studies (means and rank order)

	Model	Inspire	Challenge	Enable	Encourage
Current study	**8.27** ^d^	7.82	**8.12**	7.62 (5)	**8.04** ^d^
Clavelle^a^	**8.39** ^d^	8.22	8.16	**8.70** ^d^ **(1)**	8.17
Porter-O’Grady^b^	**8.81** ^d^	8.51	8.50	**9.17** ^d^ **(1)**	8.61
Ross^c^	8.50	8.21	8.17	**8.77** ^d^ **(1)**	**8.61** ^d^

Bolded mean scores indicate what was reported as the top-ranked (most common) leadership practices in that study.

^a^ Comparison study *N *=* *384: TLP of chief nursing officers in Magnet^®^ Organizations (Clavelle et al., 2012).

^b^ Comparison study *N *=* *161: creating a context for excellence and innovation: comparing chief nurse executive leadership practices in Magnet and non-Magnet Hospitals (Porter-O’Grady, 2009).

^c^ Comparison study *N *=* *134: TLP of nursing leaders in professional nursing associations (Ross et al., 2014).

^d^ Mean scores indicate that the number one top-ranked leadership practice for nurses in leadership is the same among three of the studies compared. Scores for the current study are still in the lowest category on *Enable others to act* compared with all three studies in high-income countries.

However, there were important noticeable differences between studies of nurses-in-leadership in the USA and the current study. Compared with leaders in Magnet Hospitals, nurses-in-leadership in this study engaged in *Inspire* and *Enable* (which was a top practice in the USA) less frequently (*M* = <8.00) than their counterparts in the USA*. Inspire a shared vision* relates to the leader’s ability to envision the future and enlist others in a common vision. Meanwhile, *Enable others to act* relates to leaders being able to foster collaboration by building trust, and strengthening others by increasing their self-determination and developing competence (Posner, 2013). Low scores on *Enable others to act and Inspire a shared vision* are of particular concern given the current leadership expectations of the nurses-in-leadership in this context. In contexts where working conditions are consistently poor and where resources are scarce, leaders who strive to inspire and enable teams are essential to create environments for shared ideas for innovation across health systems (Den Jong and Den Hartog, 2007). More evidence is needed to understand whether the low frequency of these two practices is associated with specific norms or individual or structural barriers that may limit nurses-in-leadership from fully engaging in *Inspire* and *Encourage*. It is also likely that existing systems, procedures and workplace conditions such as mentoring, employee satisfaction and morale, which were not considered in this study, have greater influence on these two practices.

A second key finding was that followers (all groups), although matching the pattern of frequency of TLP, perceived the nurse leader scores in general to be significantly less than what was self-reported. This finding is consistent with previous studies in the USA and SSA (see Ochieng Walumbwa and Lawler, 2003; Hale, 2007; Küpers and Kupers, 2008; Kean, 2011). It is difficult to ascertain whether this reflects a general sense of lack of confidence in observed leadership, or a reflection of what leaders actually do. The perception of nurses-in-leadership by followers has significant implications for how leaders emerge, how they are legitimized in their role, what power they possess, how they influence and are influenced by their followers and the strategic directions they ought to adopt to facilitate change (Kean *et al.*, 2011).

Our results suggest that nurses-in-leadership may need to explore perception gaps by their followers to strengthen these relationships. Shariff (2014) notes that the history of the poor social positioning of nurses in SSA often means that nurses-in-leadership need to work towards overcoming structural barriers by investing time and energy into rebuilding the image of nursing, starting with the people they are in leadership or working with. Nurses-in-leadership act as a critical link to the front-line managers and staff, as well as the senior management groups who oversee the entire organization. The majority of nurses-in-leadership in the study ranked themselves as either lower-level (front-line managers) or middle-level management, and were responsible for forming teams with, on average, 11–35 staff nurses. This suggests that as front-line managers, they straddle the responsibility of direct supervision of staff and patients, as well as taking responsibility for entire units (Banaszak-Holl *et al.*, 2011). The leadership burden may prove enormous, particularly in the context of lacking supportive structures, and can substantially influence leader–follower perceptions as well as jeopardize leaders' ability to sustain on-going engagement in relationship building.

A third key finding in this study was the relationship between OC (*Involvement*, *Consistency*, *Adaptability*, *Mission* and *Centralization*) and self-reported TLP where the hospital type was a marker for a specific sub-culture that was associated with leadership scores. Nurses-in-leadership in the public hospital reported higher scores than those working in the PFP and PNFP hospital types. This finding differed from Porter-O’Grady’s (2009) study in the USA which compared TLP of nurse-leaders in Magnet and non-Magnet hospitals (LPI-Self) and found no statistically significant differences. Consistent with findings from other studies (House *et al.*, 2004; Carney, 2006; Seren and Baykal, 2007), our results suggest that OC is significantly associated with TLP of nurses-in-leadership in Uganda. For example, an increase in mission culture had a significant and positive influence on the frequency of use of *Inspire*, C*hallenge*, *Enable* and *Encourage*, which is an important observation since nurses-in-leadership reported lower scores on two of these subscales. This highlights an intervention opportunity that could potentially improve leadership performance for nurses-in-leadership in this context. Involvement culture was also positively associated with increased use of *Challenge* and *Encourage* leadership practices. Fostering aspects of OC that enable and reinforce a sense of direction and that clearly delineate performance expectation can facilitate greater use of these specific TLP. A leadership environment that encourages personal growth, involvement in day-to-day decisions, a sense of direction and clear performance expectations is also likely to enable leaders in inspiring others, leading change initiatives, fostering collaboration and yielding greater leadership outcomes (Cowden *et al.*, 2011). The history of exclusion of nurses from strategic leadership and policy may be attributed to long-standing internal structures that impose restrictions of what leaders can and cannot do (Shariff, 2014). However, the renewed focus on nursing leadership provides the needed incentive for nurses-in-leadership to offer unique and contextualized perspectives on their own struggles and to help foster the needed shift in leadership strategy.

### Study limitations

Despite its many strengths, this study has limitations that should be considered in interpreting its results. First, this study used a cross-sectional design, and thus we were unable to assess how leadership practices may change over time. Second, we had limited information to assess what factors of certain hospital types could further explain differences in leadership scores. Qualitative approaches might be useful to explore nuances of leadership and organizational factors missed through quantitative measures. Last, we used a non-probability convenience sample of leaders and followers in five hospitals in a major urban centre. Although the data provide novel insights into nursing leadership practices, future studies with expanded and representative samples of nurses are warranted.

## Conclusion

Health care organizations in SSA need nurse-leaders who are able to *Model the way*, *Inspire a shared vision, Challenge the process, Enable others* and *Encourage the heart*. Leaders who use these practices frequently have the capacity to foster higher performance, follower and client satisfaction, loyalty and commitment, motivation and involvement (Kouzes, 2011). Engaging nurses-in-leadership requires an understanding of their preparedness and experience. Persisting limitations and exclusion of nurses in policy processes need to be underpinned by appropriate assessment of leadership effectiveness (Juma *et al.*, 2014; Kunaviktikul, 2014; Shariff, 2014). The new focus on nurses-in-leadership in SSA, and in Uganda in particular, brings to the forefront the urgency of action, and requires an empowered group of nurses who can substantially motivate themselves and others, as well as health system reform efforts. To succeed, nurses-in-leadership and policy makers need to understand the current leadership capacities and gaps, and what constitutes an enabling leadership environment. Our findings thus serve as a foundation for future inquiry into understanding not only the level of leadership ability but also the conditions that may contribute to nursing leadership effectiveness in SSA. Given that evidence regarding leadership practices of nurses is lacking, we believe a reasonable recommendation is for policy makers to consider available evidence to support ongoing advocacy for the inclusion of nurses in the policy making process and appropriate investments in nursing leadership development.
